# Left Ventricular Hypertrophy Is Associated with Diastolic Filling Alterations in Normotensive Offspring of Hypertensive Nigerians

**DOI:** 10.5402/2012/256738

**Published:** 2012-10-30

**Authors:** P. M. Kolo, E. O. Sanya, A. B. Omotoso, A. Soladoye, J. A. Ogunmodede

**Affiliations:** ^1^Department of Medicine, University of Ilorin, PMB 1515, Ilorin, Nigeria; ^2^Department of Physiology, University of Ilorin, PMB 1515, Ilorin, Nigeria; ^3^Department of Medicine, University of Ilorin Teaching Hospital, PMB 1459, Ilorin, Nigeria

## Abstract

Contribution of left ventricular diastolic dysfunction to adverse events in patients with cardiovascular diseases is increasingly being recognized and individuals with pedigree for hypertension are thought to exhibit anatomic and or functional changes in their left ventricle before they become hypertensive. This study aimed at characterizing left ventricular diastolic function in normotensive offspring of hypertensive Nigerians. Sixty-five offspring of hypertensive parents aged 15–25 years (subjects) with 65-age and sex-matched offspring of normotensive parents (controls) were studied for early makers of hypertensive cardiovascular disease using Doppler echocardiogram. Mean mitral E velocity was reduced (*P* = 0.01) in the subjects (73.3 ± 12.6 cm/s) compared with the controls (80.2 ± 22.5 cm/s). Similarly, mean S velocity of pulmonary venous flow was lower (*P* = 0.01) in the subjects than in the controls. Left atrial dimension and mitral E/A ratio in the subjects with left ventricular hypertrophy were higher (*P* = 0.002, 0.004 respectively) than in the subjects without this abnormality. We concluded that normotensive offspring of hypertensive Nigerians showed early alterations in indexes of left ventricular diastolic filling and these abnormalities were exaggerated in the presence of left ventricular hypertrophy.

## 1. Introduction

Left ventricular diastolic dysfunction (DD) is a common problem in adult patients with systemic hypertension with prevalence ranging between 46 and 83% [1, 2]. The presence of left ventricular DD contributes significantly to morbidity and mortality in individuals with cardiovascular diseases [3]. However, the development of left ventricular DD in the time course of hypertensive cardiovascular complications has remained a matter of controversy. Some authors have suggested left ventricular DD to be the earliest manifestation of hypertensive cardiovascular disease and antedate left ventricular structural alterations [4, 5]. On the other hand, a strong association had been shown between left ventricular hypertrophy (LVH) and left ventricular DD in hypertensive individuals [6]. Similarly, studies in OHP that evaluated structural and/or functional changes in left ventricle had yielded conflicting results [7, 8]. In one study by Piccirillo et al., abnormalities of left ventricular diastolic function were similar in OHP and that obtained from a hypertensive population [7]. In contrast to report of Piccirillo et al., Aeschbacher and his group did not find any difference in parameters of left ventricular diastolic function between OHP and offspring of ONP at baseline [8]. Interestingly, after a 5-year followup of their study cohort, left ventricular DD was found to be the earliest detectable cardiac abnormality in offspring of hypertensive individuals independent of LVH [9]. The present study aimed at characterizing left ventricular diastolic function in normotensive offspring of hypertensive parents and to determine its association with LVH. 

## 2. Materials and Methods

A random sample of hypertensive patients attending our cardiovascular clinic was conducted. The selected patients were requested to bring one of their eligible children between the ages of 15 and 25 years for assessment (OHP group). The same procedure was employed to select age- and sex-matched children without parental hypertension (ONP) from the Medical Outpatient Department to serve as controls. Ethical approval was obtained from the Ethics and Research Committee of the hospital and an informed consent was obtained from each participant. 

All individuals who were pregnant, on cardio active drugs, had cardio-pulmonary disease; and those with organic heart murmur and renal bruit were not eligible to participate. Similarly, those with structural heart disease and poor echocardiographic window were excluded from the study. A thorough history to exclude cardiovascular disease in the participants was taken and anthropometric parameters, blood pressure and other relevant clinical examination were performed on all subjects. Blood pressure was measured at the left arm in a comfortable position after about 10 minutes rest using an appropriate cuff. However, if the blood pressure is equal or greater than 10 mmHg at the right hand, the latter blood pressure was used as the blood pressure of the subjects. An average of three measurements was used as the blood pressure. Individuals with blood pressure ≥140/90 mmHg or on anti-hypertensive drugs were also excluded. Electrocardiogram was done in standard position and subsequently echocardiogram. Two-dimensional and M-mode echocardiograms were performed on all participants and measurements were taken according to the recommendations of the American Society of Echocardiography (ASE) [10]. Left ventricular mass (LVM), left ventricular mass index (LVMI) and relative wall thickness (RWT) were determined as previously described elsewhere [11]. 

Left ventricular diastolic function was assessed using Doppler echocardiography which was performed at the apical 4-chamber echocardiographic view. With the sample volume placed at the tips of mitral valve leaflets, the early diastolic (E wave), and the atrial contraction (A wave) Doppler velocity envelopes were obtained. The early to late diastolic peak velocity ratio (E/A), the flow velocity integral for both early (E1) and atrial (A1) filling waves, and the ratio of flow velocity integral of early-to-late filling (E1/A1) were used to assess diastolic function [12]. Other parameters included the rate of decrease of early diastolic flow (EF slope), deceleration time of mitral E velocity curve, Isovolumic relaxation time (IVRT), and pulmonary venous flow velocity curve. Diastolic dysfunction was classified into; impaired relaxation, pseudonormalization, and restrictive left ventricular filling. Impaired relaxation was defined as a reduced E velocity, increased left atrial contribution to left ventricular filling, reversal of the normal E/A ratio (<1), prolongation of deceleration time (>240 ms), and IVRT (>120 ms). While pseudonormal pattern was defined as left atrial enlargement, abnormal pulmonary venous waves in the presence of normal E/A ratio, restrictive pattern was defined as increased mitral peak E velocity with reduction of A velocity (E/A > 2) with shortening of the deceleration time (<160 ms) and IVRT (<70 ms) [12]. 

### 2.1. Statistical Analysis

Statistical analysis was performed using the SPSS Version 15 and numerical values were presented as mean ± standard deviation. Student *t*-test was used to compare means of continuous variables while chi-square test was used to compare means of proportions. Subanalysis of OHP based on whether LVH was present or not was done. Clinical and echocardiographic parameters of OHP with LVH were compared with those without LVH. Test of correlation was done using both Pearson and Spearman's rank correlation methods. A statistically significant association was taken at *P* < 0.05.

## 3. Results

Sixty-five OHP with 65-age and sex-matched ONP as subjects and controls, respectively, were studied. Mean ages in the two groups were similar (*P* > 0.05).

The anthropometric parameters showed that the body mass index, waist, and hip circumference; pulse rate, systolic and diastolic blood pressures were similar in the two groups as shown in Table 1. Two-dimensional echocardiogram showed right ventricular dimension in diastole, left ventricular dimension in diastole and systole to be similar between the subject and control groups. Similarly, left atrial and aortic root dimensions were not different between the two groups. However, the left ventricular posterior wall thickness in OHP was significantly higher (*P* = 0.001) than that of ONP. Similarly, the left ventricular mass, left ventricular mass index, and relative wall thickness were significantly higher in the subjects than controls (*P* = 0.01, 0.01, and 0.001, resp.). 

Doppler echocardiographic assessment of the subjects is presented in Table 2. Mean peak aortic and pulmonary artery systolic velocities were similar in both OHP and ONP groups. Mean mitral E velocity envelope in the OHP (73.3 ± 12.6 cm/s) was significantly lower than in ONP (80.2 ± 22.5 cm/s), *P* = 0.01. However, mean mitral A velocity envelope and E/A ratio were similar in both groups (*P* = 0.25, 0.7, resp.). Similarly, mitral E velocity deceleration time and isovolumic relaxation time were not different between the two groups (*P* = 0.6 and 0.9, resp.). Mean S velocity envelope of pulmonary venous flow was significantly lower (*P* = 0.01) in the subjects than in the control, although both were within normal limits in the two groups. On the other hand, mean D velocity and atrial reversal velocities were similar between the two groups (*P* = 0.087 and 0.9). Overall, patterns of left ventricular diastolic function were similar between the two groups. None of the subjects and controls had impaired relaxation, pseudonormal and restrictive patterns. 

Comparison of OHP with and without left ventricular hypertrophy is displayed in Table 3. Mean BMI of those with LVH was 23.3 ± 1.6 g/m^2^ which was higher than mean of those without LVH (21.8 ± 3.2 g/m^2^), *P* = 0.03. Similarly, waist circumference in those with LVH was significantly higher than the mean of those without LVH (*P* = 0.045). Mean left atrial dimension in OHP with LVH was 3.39 ± 0.3 cm which was higher than 2.99 ± 0.45 cm found in OHP without LVH, *P* = 0.002. While mitral A velocity was significantly lower in those with LVH compared with mean of OHP without LVH (*P* = 0.004), mitral E/A ratio was higher in the former than the latter (*P* = 0.0).

Correlates of mitral E, A velocities, and E/A ratio are shown in Table 4. Mitral E velocity correlated negatively with both echocardographic LVH and aortic root dimension. Similarly, negative correlation was observed between mitral E velocity and age. While mitral A velocity correlated positively with echocardiographic LVH, negative associated was seen between mitral A velocity and aortic root dimension. 

## 4. Discussion

This study shows significant reduction in some of the indexes of left ventricular diastolic function in OHP when compared with ONP. Both mitral E velocity and S wave of pulmonary venous flow were significantly lower in OHP than ONP. Although, the differences in these parameters between the two groups of offspring were small, they may indicate subtle alterations in the left ventricular diastolic function in the former, with filling pressures tending towards the earliest pattern of left ventricular DD seen in systemic hypertension. The pathophysiological mechanisms underlying these early alterations are unknown. However, elevated endogenous ouabain has been suggested as a possible mechanism and its plasma level had been found to correlate well with indexes of diastolic dysfunction in offspring of hypertensive parents [13].

Abnormalities of left ventricular filling pressures observed in our study may be partly due to higher left ventricular mass in OHP compared with ONP [14]. All other indexes of left ventricular diastolic function including mitral A velocity, mitral E/A ratio, deceleration time, and isovolumic relaxation time were similar between the two groups. These findings concur with some of the results of earlier studies in offspring with genetic predisposition to hypertension [15, 16]. In a study by Graettinger et al. among young US adults with family history of hypertension, alterations in mitral A velocity were found [15]. Left ventricular diastolic function in that study while still normal shifted towards the pattern of left ventricular filling observed in hypertensive individuals. 

Generally, left ventricular diastolic filling patterns in patients with systemic hypertension include normal, impaired relaxation, pseudonormal and restrictive patterns [17]. All the subjects and controls in the present study had normal pattern of left ventricular diastolic function. None of the subjects and controls had impaired relaxation, pseudonormal and restrictive patterns. 

The results of the present study also showed that OHP with LVH had higher BMI, WC, and DBP than OHP without LVH. These findings clearly demonstrated the effects of larger body size on blood pressure, left ventricular structure, and filling. The mitral E/A ratio in OHP with LVH was greater than 2 which was suggestive of restrictive patterns of left ventricular filling unlike normal pattern seen in OHP without LVH. More importantly, left atrial dimension which is a reflection of left ventricular filling pressure was significantly large in those with LVH than those without LVH. This indicates higher left ventricular filling pressure in OHP with LVH than those without LVH. The influence of LVH on left ventricular diastolic function had been shown by some studies [18, 19]. It has been suggested that normotensive OHP do not exhibit alterations in left ventricular diastolic function in the absence of LVH [8]. Our study showed that the presence of LVH increases the prevalence of abnormalities of left ventricular filling pressures. Similarly, this relationship was further supported by findings on electrocardiogram in our study. While significant positive correlation was seen between electrocardiographic LVH and mitral A velocity, a negative association was demonstrated with mitral E/A ratio in OHP. Consequently, lifestyle changes to achieve normal body weight, BMI, and reduction in LVM in OHP will go a long way to delay or prevent onset and progression of alterations in left ventricular diastolic function.

Negative correlation was observed between mitral E velocity envelope and age. This pattern is similar to what is obtained in older population of both normotensive and hypertensive individuals [20, 21]. On the other hand, negative correlation was seen between mitral A velocity envelope and E/A ratio with aortic root dimension.

In conclusion, normotensive offspring of hypertensive Nigerians showed early alterations in some of the indexes of left ventricular diastolic filling and these abnormalities are exaggerated in the presence of LVH. We recommend early and appropriate lifestyles modification in OHP especially those with LVH.

## Figures and Tables

**Table 1:** Clinical and echocardiographic parameters in the study group.

Dimension	Subjects	Controls	*P* value
	Mean (SD)	Mean (SD)	
Number	65	65	
Age (years)	21.6 (0.29)	21.7 (0.26)	0.88
Body mass index (kg/m^2^)	22.1 (3.0)	21.7 (2.8)	0.41
WC (cm)	80.4 (8.9)	78.3 (7.1)	0.22
Pulse rate (beats/min)	76.8 (11.6)	74.5 (9.7)	0.32
SBP (mmHg)	118.2 (9.0)	115.9 (8.8)	0.14
DBP (mmHg)	75.6 (7.6)	72.8 (8.8)	0.06
RVd (mm)	13.3 (4.5)	14.3 (3.6)	0.14
IVSd (mm)	11.2 (2.4)	11.2 (2.7)	0.97
PWd (mm)	9.7 (2.3)	8.5 (1.8)	0.001*
LVDd (mm)	45.6 (5.3)	45.4 (5.8)	0.8
LVDs (mm)	29.2 (6.5)	29.1 (4.8)	0.92
Ejection fraction (%)	66.8 (8.4)	65.5 (8.8)	0.4
LVM (g)	196.9 (63.8)	175.2 (58.8)	0.01*
LVMI (g/m^2^)	114.5 (32.6)	103.2 (26.1)	0.01*
AOD (mm)	27.3 (2.9)	27.2 (3.0)	0.78
LAD (mm)	30.7 (4.5)	30.8 (5.0)	0.88
RWT	0.43 (0.11)	0.38 (0.09)	0.001*

Key: WC: waist circumference, RVd: right ventricular dimension, IVSd: interventricular septum in diastole, PWd: posterior wall in diastole, LVDd: left ventricular dimension in diastole, LVDs: left ventricular dimension in systole, LVM: left ventricular mass, LVMI: left ventricular mass index, AOD: aortic dimension, LAD: left atrial dimension, and RWT: relative wall thickness. *Statistically significant.

**Table 2:** Doppler echocardiographic indices in the subjects and the controls.

Indices	Subjects	Controls	*P* value
	Mean (SD)	Mean (SD)	
Peak aortic velocity (cm/s)	99.7 (27.1)	100.8 (17.6)	0.8
Peak pulmonary velocity (cm/s)	95.8 (21.3)	94.0 (15.0)	0.6
Mitral inflow velocities			
E velocity (cm/s)	73.3 (12.6)	80.2 (22.5)	0.01*
A velocity (cm/s)	42.8 (9.8)	45.1 (12.8)	0.25
E/A Ratio	1.78 (0.43)	1.82 (0.49)	0.7
Deceleration time (ms)	236.2 (99)	247.6 (122.5)	0.6
Isovolumic relaxation time (ms)	98.7 (16.5)	98.8 (17.1)	0.9
Pulmonary venous flow			
S wave (cm/s)	49.5 (8.5)	55.8 (9.5)	0.01*
D wave (cm/s)	37.3 (11.7)	43.5 (10.7)	0.087
Atrial reversal wave (cm/s)	25.3 (6.0)	25.0 (5.7)	0.9

*Statistically significant.

**Table 3:** Comparison of offspring of hypertensive parents with and without left ventricular hypertrophy.

Parameter	LVH +ve	LVH −ve	*P* value
	Mean (SD)	Mean (SD)	
Number	12	53	
Age (years)	21.4 (1.7)	21.7 (2.5)	0.7
BMI (Kg/m^2^)	23.3 (1.6)	21.8 (3.2)	0.03*
WC (cm)	85.3 (8.0)	78.8 (8.7)	0.045*
Pulse rate (beats/min)	77.4 (10.3)	75.8 (11.8)	0.22
SBP (mmHg)	122.5 (8.7)	117.3 (8.9)	0.068
DBP (mmHg)	79.6 (5.0)	74.7 (7.9)	0.01*
LAD (cm)	3.39 (0.3)	2.99 (0.45)	0.002*
Ejection fraction (%)	61.3 (5.1)	68.0 (8.5)	0.01*
Mitral E velocity (cm/s)	73.7 (12.4)	73.2 (12.7)	0.9
Mitral A velocity (cm/s)	36.3 (7.0)	44.3 (9.8)	0.004*
Mitral E/A ratio	2.1 (0.4)	1.7 (0.4)	0.01*
Mitral DT (ms)	263 (111)	229 (96.1)	0.4
IVRT (ms)	105.3 (4.6)	97.8 (7.5)	0.13
PVF S wave (cm/s)	48.4 (8.2)	49.6 (8.7)	0.24
PVF D wave (cm/s)	46.0 (9.1)	36.7 (11.9)	0.01
PVF AR wave (cm/s)	21.3 (5.6)	25.6 (6.0)	0.06

Key: BMI: body mass index, WC: waist circumference, SBP: systolic blood pressure, DBP: diastolic blood pressure, LAD: left atrial dimension, DT: deceleration time, IVRT: isovolumic relaxation time, and PVF: pulmonary venous flow. *Statistically significant.

**Table 4:** Correlates of indices of left ventricular diastolic function in offspring of hypertensive parents.

Parameters	E velocity	A velocity	E/A ratio
	(*R*, *P* value)	(*R*, *P* value)	(*R*, *P* value)
Age	−0.279 (0.024)*	−0.01 (0.9)	−0.081 (0.5)
BMI	0.093 (0.46)	0.032 (0.8)	0.051 (0.69)
LVHe	−0.024 (0.85)	0.298 (0.016)*	−0.325 (0.008)*
WC	−0.116 (0.5)	−0.224 (0.18)	0.052 (0.8)
AOD	−0.139 (0.3)	−0.287 (0.02)*	−0.255 (0.04)*
LAD	−0.008 (0.95)	0.072 (0.57)	−0.006 (0.96)

Key: BMI: body mass index, LVHE: electrocardiographic left ventricular hypertrophy, WC: waist circumference, AOD: aortic dimension, *R*: is the coefficient of correlation, and LAD: left atrial dimension. *Statistically significant.
